# Potassium *N*-chloro-*o*-toluene­sulfonamidate monohydrate

**DOI:** 10.1107/S1600536811023555

**Published:** 2011-06-22

**Authors:** B. Thimme Gowda, Sabine Foro, K. Shakuntala

**Affiliations:** a Department of Chemistry, Mangalore University, Mangalagangotri 574 199, Mangalore, India; b Institute of Materials Science, Darmstadt University of Technology, Petersenstrasse 23, D-64287 Darmstadt, Germany

## Abstract

In the crystal structure of the title compound, K^+^·C_7_H_7_ClNO_2_S^−^·H_2_O, the K^+^ ion is hepta­coordinated by two O atoms from water mol­ecules, four sulfonyl O atoms and the Cl atom of the anion. The S—N distance of 1.584 (3) Å is consistent with an S—N double bond. In the crystal, anions are connected by K^+^ cations into layers parallel to the *ab* plane. The water mol­ecules are coordinated to the K^+^ cations and are additionally linked by inter­molecular O—H⋯Cl and O—H⋯N hydrogen bonding.

## Related literature

For our studies of the effect of substituents on the structures of *N*-haloaryl­sulfonamides, see: Gowda *et al.* (2009 ▶, 2011**a* ▶,b* ▶); and on the oxidative strengths of *N*-halolaryl­sulfonamides, see: Gowda & Kumar (2003 ▶); Usha & Gowda (2006 ▶). For similar structures, see: George *et al.* (2000 ▶); Olmstead & Power (1986 ▶). For the preparation of the title compound, see: Jyothi & Gowda (2004 ▶).
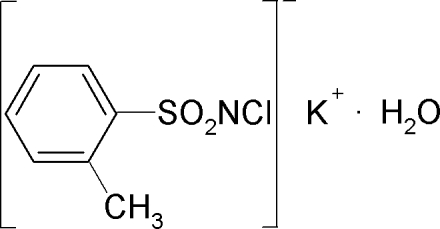


## Experimental

### 

#### Crystal data

K^+^·C_7_H_7_ClNO_2_S^−^·H_2_O
*M* *_r_* = 261.76
Orthorhombic, 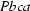

*a* = 11.4968 (9) Å
*b* = 6.7990 (5) Å
*c* = 26.883 (2) Å
*V* = 2101.4 (3) Å^3^
*Z* = 8
Mo *K*α radiation
μ = 0.94 mm^−1^
*T* = 293 K
0.42 × 0.40 × 0.30 mm

#### Data collection

Oxford Diffraction Xcalibur diffractometer with a Sapphire CCD detector
Absorption correction: multi-scan (*CrysAlis RED*; Oxford Diffraction, 2009 ▶) *T*_min_ = 0.694, *T*_max_ = 0.766
4396 measured reflections
2150 independent reflections
1992 reflections with *I* > 2σ(*I*)
*R*_int_ = 0.017

#### Refinement

*R*[*F* ^2^ > 2σ(*F* ^2^)] = 0.044
*wR*(*F* ^2^) = 0.123
*S* = 0.92
2150 reflections
134 parameters
4 restraints
H atoms treated by a mixture of independent and constrained refinement
Δρ_max_ = 0.42 e Å^−3^
Δρ_min_ = −0.59 e Å^−3^

### 

Data collection: *CrysAlis CCD* (Oxford Diffraction, 2009 ▶); cell refinement: *CrysAlis RED* (Oxford Diffraction, 2009 ▶); data reduction: *CrysAlis RED*; program(s) used to solve structure: *SHELXS97* (Sheldrick, 2008 ▶); program(s) used to refine structure: *SHELXL97* (Sheldrick, 2008 ▶); molecular graphics: *PLATON* (Spek, 2009 ▶); software used to prepare material for publication: *SHELXL97*.

## Supplementary Material

Crystal structure: contains datablock(s) I, global. DOI: 10.1107/S1600536811023555/nc2234sup1.cif

Structure factors: contains datablock(s) I. DOI: 10.1107/S1600536811023555/nc2234Isup2.hkl

Additional supplementary materials:  crystallographic information; 3D view; checkCIF report

## Figures and Tables

**Table 1:** Hydrogen-bond geometry (Å, °)

*D*—H⋯*A*	*D*—H	H⋯*A*	*D*⋯*A*	*D*—H⋯*A*
O3—H31⋯Cl1^i^	0.85 (1)	2.74 (2)	3.568 (3)	166 (4)
O3—H32⋯N1^ii^	0.85 (1)	2.08 (1)	2.909 (4)	167 (3)

Symmetry codes: (i) 
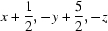
; (ii) 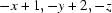.

**Fig. 1.**
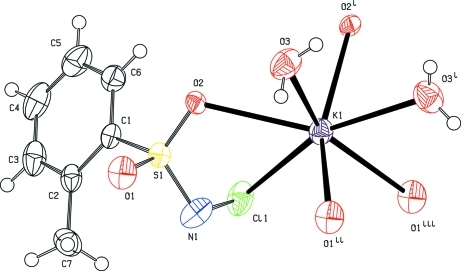
Molecular structure of the title compound showing the coordination geometry of the K+ cations with labelling and displacement ellipsoids drawn at the 50% probability level. Symmetry codes: (i) -x + 3/2, y + 1/2, z; (ii) -x + 1, -y + 2, -z; (iii) x, y + 1, z.

**Fig. 2.**
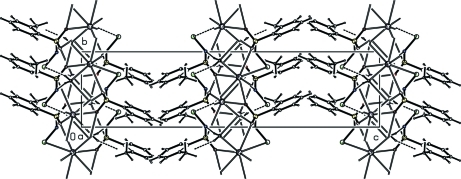
Crystal structure of the title compound with view in the direction of the a axis and hydrogen bonding drawn as dashed lines.

## supplementary crystallographic information

### Comment

The crystal structure of the title compound (I) was determined as a part of a project to explore the substituent effects and the effect of replacing sodium ions by potassium ions on the solid state structures of *N*-halo-arylsulfonamides  (Gowda & Kumar, 2003; Usha & Gowda, 2006, Gowda *et al.*, 2009, 2011**a*,b*). The structure resembles those of potassium *N*, 2-dichloro-benzenesulfonamidate sesquihydrate (II)(Gowda *et al.*, 2011*a*), potassium *N*-bromo, *o*-toluenesulfonamidate sesquihydrate (Gowda *et al.*, 2011*b*) and sodium *N*-chloro, *o*-toluenesulfonamidate sesquihydrate (IV) (Gowda *et al.*, 2009) and other sodium *N*-chloro-aryl- sulfonamidates (George *et al.*, 2000; Olmstead & Power, 1986).

In the crystal structure of the title compound the K^+^ ion is hepta coordinated by two O atoms from water molecules, four sulfonyl O atoms and one Cl atom of the *N*-chloro,*o*-toluenesulfonamidate anions (Fig. 1). This coordination geometry is different from that in II and III, in which the potassium cations are hepta coordinated by three O atoms from water molecules and by four sulfonyl O atoms and and in III, in which the cations are octahedral coordinated.

The S—N distance of 1.584 (3)Å is consistent with an S—N double bond and is in agreement with the observed values of 1.582 (2)Å in II, 1.577 (5)Å in III and 1.590 (2) Å in IV.

The crystal structure comprises sheets parallel to the *ab* plane (Fig. 2). The molecular packing is additionally stabilized by O—H···Cl and O—H···N hydrogen bonds (Table 1).

### Experimental

The title compound was prepared by the method similar to that reported in literature (Jyothi & Gowda, 2004). *o*-Toluenesulfonamide (2 g) was dissolved in hot aqueous solution (70° C) of 5 *M* KOH (40 ml). The resulting solution was filtered and Chlorine gas was passed through the clear solution of *o*-toluenesulfonamide in KOH to obtain the title compound. It was filtered under suction, quickly washed with a minimum quantity of ice cold water and dried. The purity of the compound was checked by determining its melting point (155° C). Colourless prisms of the compound were obtained from its aqueous solution at room temperature.

### Refinement

The water H atoms were located in difference map and were refined with O—H distance restrained to 0.85 (2) Å and H—H distance restrained to 1.365 Å. The other H atoms were positioned with idealized geometry using a riding model with the aromatic C—H = 0.93 Å and methyl C—H = 0.96 Å. All H atoms were refined with isotropic displacement parameters (set to 1.2 times of the *U*_eq_ of the parent atom).

### Crystal data

**Table:**

K^+^·C_7_H_7_ClNO_2_S^−^·H_2_O	*F*(000) = 1072
*M**_r_* = 261.76	*D*_x_ = 1.655 Mg m^−^^3^
Orthorhombic, *P**b**c**a*	Mo *K*α radiation, λ = 0.71073 Å
Hall symbol: -P 2ac 2ab	Cell parameters from 2737 reflections
*a* = 11.4968 (9) Å	θ = 3.0–27.7°
*b* = 6.7990 (5) Å	µ = 0.94 mm^−^^1^
*c* = 26.883 (2) Å	*T* = 293 K
*V* = 2101.4 (3) Å^3^	Prism, colourless
*Z* = 8	0.42 × 0.40 × 0.30 mm

### Data collection

**Table:**

Oxford Diffraction Xcalibur diffractometer with a Sapphire CCD detector	2150 independent reflections
Radiation source: fine-focus sealed tube	1992 reflections with *I* > 2σ(*I*)
graphite	*R*_int_ = 0.017
Rotation method data acquisition using ω scans	θ_max_ = 26.4°, θ_min_ = 3.5°
Absorption correction: multi-scan (*CrysAlis RED*; Oxford Diffraction, 2009)	*h* = −2→14
*T*_min_ = 0.694, *T*_max_ = 0.766	*k* = −8→5
4396 measured reflections	*l* = −33→14

### Refinement

**Table:**

Refinement on *F*^2^	Primary atom site location: structure-invariant direct methods
Least-squares matrix: full	Secondary atom site location: difference Fourier map
*R*[*F*^2^ > 2σ(*F*^2^)] = 0.044	Hydrogen site location: inferred from neighbouring sites
*wR*(*F*^2^) = 0.123	H atoms treated by a mixture of independent and constrained refinement
*S* = 0.92	*w* = 1/[σ^2^(*F*_o_^2^) + (0.0717*P*)^2^ + 5.301*P*] where *P* = (*F*_o_^2^ + 2*F*_c_^2^)/3
2150 reflections	(Δ/σ)_max_ = 0.001
134 parameters	Δρ_max_ = 0.42 e Å^−^^3^
4 restraints	Δρ_min_ = −0.59 e Å^−^^3^

### Special details

**Table:**

Geometry. All e.s.d.'s (except the e.s.d. in the dihedral angle between two l.s. planes) are estimated using the full covariance matrix. The cell e.s.d.'s are taken into account individually in the estimation of e.s.d.'s in distances, angles and torsion angles; correlations between e.s.d.'s in cell parameters are only used when they are defined by crystal symmetry. An approximate (isotropic) treatment of cell e.s.d.'s is used for estimating e.s.d.'s involving l.s. planes.
Refinement. Refinement of *F*^2^ against ALL reflections. The weighted *R*-factor *wR* and goodness of fit *S* are based on *F*^2^, conventional *R*-factors *R* are based on *F*, with *F* set to zero for negative *F*^2^. The threshold expression of *F*^2^ > σ(*F*^2^) is used only for calculating *R*-factors(gt) *etc*. and is not relevant to the choice of reflections for refinement. *R*-factors based on *F*^2^ are statistically about twice as large as those based on *F*, and *R*- factors based on ALL data will be even larger.

### Fractional atomic coordinates and isotropic or equivalent isotropic displacement parameters (Å^2^)

**Table:**

	*x*	*y*	*z*	*U*_iso_*/*U*_eq_	
K1	0.63387 (6)	1.33711 (10)	0.02856 (2)	0.0327 (2)	
Cl1	0.47408 (9)	1.20077 (12)	0.12455 (3)	0.0481 (3)	
S1	0.55243 (6)	0.85908 (10)	0.08371 (2)	0.0260 (2)	
O1	0.5175 (2)	0.6996 (3)	0.05138 (8)	0.0412 (5)	
N1	0.4458 (2)	1.0067 (4)	0.08324 (10)	0.0368 (6)	
O3	0.7703 (2)	1.1144 (4)	−0.03605 (9)	0.0494 (6)	
H31	0.817 (3)	1.179 (6)	−0.0541 (12)	0.059*	
H32	0.713 (2)	1.084 (7)	−0.0541 (12)	0.059*	
O2	0.66241 (18)	0.9505 (3)	0.07163 (8)	0.0367 (5)	
C1	0.5708 (2)	0.7601 (4)	0.14466 (10)	0.0251 (5)	
C2	0.4782 (3)	0.6704 (4)	0.17010 (11)	0.0305 (6)	
C3	0.5030 (3)	0.5932 (5)	0.21709 (12)	0.0426 (8)	
H3	0.4441	0.5301	0.2346	0.051*	
C4	0.6116 (4)	0.6073 (5)	0.23828 (12)	0.0492 (9)	
H4	0.6249	0.5548	0.2697	0.059*	
C5	0.7001 (3)	0.6985 (5)	0.21318 (13)	0.0466 (8)	
H5	0.7732	0.7096	0.2277	0.056*	
C6	0.6803 (3)	0.7742 (5)	0.16600 (11)	0.0348 (6)	
H6	0.7406	0.8345	0.1487	0.042*	
C7	0.3566 (3)	0.6577 (5)	0.15053 (14)	0.0427 (8)	
H7A	0.3582	0.6144	0.1165	0.051*	
H7B	0.3205	0.7849	0.1524	0.051*	
H7C	0.3129	0.5657	0.1701	0.051*	

### Atomic displacement parameters (Å^2^)

**Table:**

	*U*^11^	*U*^22^	*U*^33^	*U*^12^	*U*^13^	*U*^23^
K1	0.0370 (4)	0.0316 (4)	0.0294 (3)	−0.0002 (3)	−0.0028 (2)	0.0026 (3)
Cl1	0.0630 (6)	0.0315 (4)	0.0497 (5)	0.0118 (4)	0.0137 (4)	−0.0022 (3)
S1	0.0319 (4)	0.0261 (4)	0.0199 (3)	0.0016 (3)	0.0016 (2)	0.0001 (2)
O1	0.0564 (14)	0.0378 (12)	0.0293 (11)	−0.0012 (11)	−0.0026 (10)	−0.0105 (10)
N1	0.0396 (14)	0.0365 (14)	0.0344 (13)	0.0095 (11)	−0.0050 (10)	0.0007 (11)
O3	0.0535 (15)	0.0560 (16)	0.0387 (12)	0.0129 (13)	0.0017 (11)	0.0097 (12)
O2	0.0377 (10)	0.0383 (12)	0.0340 (11)	−0.0015 (8)	0.0096 (9)	0.0056 (9)
C1	0.0325 (13)	0.0201 (12)	0.0228 (12)	0.0029 (11)	−0.0005 (10)	−0.0009 (10)
C2	0.0406 (15)	0.0216 (13)	0.0293 (14)	0.0025 (11)	0.0092 (12)	−0.0011 (11)
C3	0.062 (2)	0.0311 (16)	0.0344 (16)	0.0036 (15)	0.0161 (15)	0.0062 (13)
C4	0.079 (3)	0.0414 (18)	0.0274 (15)	0.0164 (18)	−0.0037 (16)	0.0082 (14)
C5	0.057 (2)	0.0455 (19)	0.0377 (17)	0.0096 (16)	−0.0175 (15)	0.0012 (15)
C6	0.0377 (15)	0.0324 (15)	0.0343 (15)	0.0002 (13)	−0.0037 (12)	0.0013 (12)
C7	0.0375 (16)	0.0408 (18)	0.0499 (19)	−0.0067 (14)	0.0107 (14)	−0.0013 (15)

### Geometric parameters (Å, °)

**Table:**

K1—O2^i^	2.724 (2)	O3—H31	0.848 (10)
K1—O1^ii^	2.777 (2)	O3—H32	0.847 (10)
K1—O3	2.788 (3)	O2—K1^v^	2.724 (2)
K1—O3^i^	2.790 (3)	C1—C6	1.386 (4)
K1—O1^iii^	2.870 (2)	C1—C2	1.405 (4)
K1—O2	2.891 (2)	C2—C3	1.397 (4)
K1—Cl1	3.3006 (11)	C2—C7	1.497 (5)
K1—N1	3.447 (3)	C3—C4	1.375 (6)
K1—H31	3.25 (4)	C3—H3	0.9300
K1—H32	2.95 (4)	C4—C5	1.369 (6)
Cl1—N1	1.755 (3)	C4—H4	0.9300
S1—O1	1.446 (2)	C5—C6	1.388 (4)
S1—O2	1.446 (2)	C5—H5	0.9300
S1—N1	1.584 (3)	C6—H6	0.9300
S1—C1	1.784 (3)	C7—H7A	0.9600
O1—K1^ii^	2.777 (2)	C7—H7B	0.9600
O1—K1^iv^	2.870 (2)	C7—H7C	0.9600
O3—K1^v^	2.790 (3)		
			
O2^i^—K1—O1^ii^	153.30 (7)	O1—S1—O2	115.46 (14)
O2^i^—K1—O3	86.25 (8)	O1—S1—N1	104.81 (15)
O1^ii^—K1—O3	79.74 (8)	O2—S1—N1	113.75 (14)
O2^i^—K1—O3^i^	74.57 (7)	O1—S1—C1	107.58 (13)
O1^ii^—K1—O3^i^	80.01 (7)	O2—S1—C1	105.34 (13)
O3—K1—O3^i^	75.94 (5)	N1—S1—C1	109.75 (13)
O2^i^—K1—O1^iii^	93.83 (7)	S1—O1—K1^ii^	134.92 (14)
O1^ii^—K1—O1^iii^	87.16 (6)	S1—O1—K1^iv^	130.02 (14)
O3—K1—O1^iii^	149.53 (7)	K1^ii^—O1—K1^iv^	92.84 (6)
O3^i^—K1—O1^iii^	74.75 (8)	S1—N1—Cl1	109.14 (15)
O2^i^—K1—O2	89.39 (6)	S1—N1—K1	86.03 (11)
O1^ii^—K1—O2	107.42 (7)	Cl1—N1—K1	70.34 (10)
O3—K1—O2	72.04 (7)	K1—O3—K1^v^	101.62 (8)
O3^i^—K1—O2	144.97 (8)	K1—O3—H31	116 (3)
O1^iii^—K1—O2	138.41 (7)	K1^v^—O3—H31	117 (3)
O2^i^—K1—Cl1	103.04 (5)	K1—O3—H32	93 (3)
O1^ii^—K1—Cl1	103.35 (6)	K1^v^—O3—H32	120 (3)
O3—K1—Cl1	130.37 (6)	H31—O3—H32	107.3 (16)
O3^i^—K1—Cl1	153.67 (7)	S1—O2—K1^v^	136.54 (13)
O1^iii^—K1—Cl1	79.32 (5)	S1—O2—K1	112.42 (12)
O2—K1—Cl1	59.65 (4)	K1^v^—O2—K1	100.64 (7)
O2^i^—K1—N1	122.85 (7)	C6—C1—C2	121.1 (3)
O1^ii^—K1—N1	83.01 (7)	C6—C1—S1	117.5 (2)
O3—K1—N1	105.34 (8)	C2—C1—S1	121.4 (2)
O3^i^—K1—N1	162.46 (7)	C3—C2—C1	116.6 (3)
O1^iii^—K1—N1	100.15 (7)	C3—C2—C7	119.1 (3)
O2—K1—N1	46.20 (6)	C1—C2—C7	124.2 (3)
Cl1—K1—N1	30.05 (5)	C4—C3—C2	122.2 (3)
O2^i^—K1—H31	80.0 (6)	C4—C3—H3	118.9
O1^ii^—K1—H31	81.2 (6)	C2—C3—H3	118.9
O3—K1—H31	13.6 (5)	C3—C4—C5	120.1 (3)
O3^i^—K1—H31	62.7 (6)	C3—C4—H4	119.9
O1^iii^—K1—H31	137.2 (6)	C5—C4—H4	119.9
O2—K1—H31	84.2 (6)	C4—C5—C6	119.8 (3)
Cl1—K1—H31	143.4 (6)	C4—C5—H5	120.1
N1—K1—H31	118.9 (5)	C6—C5—H5	120.1
O2^i^—K1—H32	102.8 (3)	C5—C6—C1	120.1 (3)
O1^ii^—K1—H32	63.8 (5)	C5—C6—H6	120.0
O3—K1—H32	16.6 (3)	C1—C6—H6	120.0
O3^i^—K1—H32	78.7 (8)	C2—C7—H7A	109.5
O1^iii^—K1—H32	143.6 (7)	C2—C7—H7B	109.5
O2—K1—H32	74.7 (8)	H7A—C7—H7B	109.5
Cl1—K1—H32	126.5 (7)	C2—C7—H7C	109.5
N1—K1—H32	97.6 (6)	H7A—C7—H7C	109.5
H31—K1—H32	24.8 (3)	H7B—C7—H7C	109.5
N1—Cl1—K1	79.61 (10)		
			
O2^i^—K1—Cl1—N1	−135.35 (10)	N1—K1—O3—K1^v^	−57.22 (10)
O1^ii^—K1—Cl1—N1	48.70 (10)	O1—S1—O2—K1^v^	−29.4 (2)
O3—K1—Cl1—N1	−39.09 (12)	N1—S1—O2—K1^v^	−150.65 (16)
O3^i^—K1—Cl1—N1	143.14 (15)	C1—S1—O2—K1^v^	89.11 (19)
O1^iii^—K1—Cl1—N1	133.16 (10)	O1—S1—O2—K1	107.12 (14)
O2—K1—Cl1—N1	−53.88 (10)	N1—S1—O2—K1	−14.13 (17)
O2—S1—O1—K1^ii^	−99.9 (2)	C1—S1—O2—K1	−134.36 (11)
N1—S1—O1—K1^ii^	26.1 (2)	O2^i^—K1—O2—S1	147.79 (8)
C1—S1—O1—K1^ii^	142.86 (17)	O1^ii^—K1—O2—S1	−53.29 (14)
O2—S1—O1—K1^iv^	58.3 (2)	O3—K1—O2—S1	−125.95 (14)
N1—S1—O1—K1^iv^	−175.69 (16)	O3^i^—K1—O2—S1	−150.81 (12)
C1—S1—O1—K1^iv^	−58.93 (19)	O1^iii^—K1—O2—S1	52.71 (17)
O1—S1—N1—Cl1	176.31 (15)	Cl1—K1—O2—S1	42.26 (10)
O2—S1—N1—Cl1	−56.67 (19)	N1—K1—O2—S1	8.18 (10)
C1—S1—N1—Cl1	61.05 (19)	O2^i^—K1—O2—K1^v^	−60.99 (11)
O1—S1—N1—K1	−116.09 (11)	O1^ii^—K1—O2—K1^v^	97.93 (8)
O2—S1—N1—K1	10.93 (13)	O3—K1—O2—K1^v^	25.27 (8)
C1—S1—N1—K1	128.65 (10)	O3^i^—K1—O2—K1^v^	0.41 (16)
K1—Cl1—N1—S1	78.36 (14)	O1^iii^—K1—O2—K1^v^	−156.08 (8)
O2^i^—K1—N1—S1	−57.37 (13)	Cl1—K1—O2—K1^v^	−166.52 (9)
O1^ii^—K1—N1—S1	115.47 (11)	N1—K1—O2—K1^v^	159.39 (12)
O3—K1—N1—S1	38.17 (12)	O1—S1—C1—C6	119.2 (2)
O3^i^—K1—N1—S1	130.0 (2)	O2—S1—C1—C6	−4.5 (3)
O1^iii^—K1—N1—S1	−158.69 (10)	N1—S1—C1—C6	−127.3 (2)
O2—K1—N1—S1	−6.91 (9)	O1—S1—C1—C2	−60.5 (3)
Cl1—K1—N1—S1	−111.95 (14)	O2—S1—C1—C2	175.8 (2)
O2^i^—K1—N1—Cl1	54.58 (11)	N1—S1—C1—C2	53.0 (3)
O1^ii^—K1—N1—Cl1	−132.58 (10)	C6—C1—C2—C3	−1.4 (4)
O3—K1—N1—Cl1	150.12 (9)	S1—C1—C2—C3	178.3 (2)
O3^i^—K1—N1—Cl1	−118.0 (3)	C6—C1—C2—C7	177.1 (3)
O1^iii^—K1—N1—Cl1	−46.74 (10)	S1—C1—C2—C7	−3.2 (4)
O2—K1—N1—Cl1	105.04 (11)	C1—C2—C3—C4	1.5 (5)
O2^i^—K1—O3—K1^v^	65.85 (9)	C7—C2—C3—C4	−177.1 (3)
O1^ii^—K1—O3—K1^v^	−136.97 (10)	C2—C3—C4—C5	−0.3 (5)
O3^i^—K1—O3—K1^v^	140.87 (13)	C3—C4—C5—C6	−0.9 (5)
O1^iii^—K1—O3—K1^v^	157.04 (11)	C4—C5—C6—C1	1.0 (5)
O2—K1—O3—K1^v^	−24.72 (8)	C2—C1—C6—C5	0.2 (5)
Cl1—K1—O3—K1^v^	−38.10 (13)	S1—C1—C6—C5	−179.5 (3)

Symmetry codes: (i) −*x*+3/2, *y*+1/2, *z*; (ii) −*x*+1, −*y*+2, −*z*; (iii) *x*, *y*+1, *z*; (iv) *x*, *y*−1, *z*; (v) −*x*+3/2, *y*−1/2, *z*.

### Hydrogen-bond geometry (Å, °)

**Table:**

*D*—H···*A*	*D*—H	H···*A*	*D*···*A*	*D*—H···*A*
O3—H31···Cl1^vi^	0.85 (1)	2.74 (2)	3.568 (3)	166 (4)
O3—H32···N1^ii^	0.85 (1)	2.08 (1)	2.909 (4)	167 (3)

Symmetry codes: (vi) *x*+1/2, −*y*+5/2, −*z*; (ii) −*x*+1, −*y*+2, −*z*.
